# DNA Microarray Characterization of Pathogens Associated with Sexually Transmitted Diseases

**DOI:** 10.1371/journal.pone.0133927

**Published:** 2015-07-24

**Authors:** Boyang Cao, Suwei Wang, Zhenyang Tian, Pinliang Hu, Lu Feng, Lei Wang

**Affiliations:** 1 Key Laboratory of Molecular Microbiology and Technology of the Ministry of Education, TEDA College, Nankai University, Tianjin, P. R. China; 2 TEDA Institute of Biological Sciences and Biotechnology, Nankai University, Tianjin, P. R. China; 3 Tianjin Research Center for Functional Genomics and Biochips, TEDA College, Nankai University, Tianjin, P. R. China; 4 Tianjin Key Laboratory of Microbial Functional Genomics, TEDA College, Nankai University, Tianjin, P. R. China; University of Georgia, UNITED STATES

## Abstract

This study established a multiplex PCR-based microarray to detect simultaneously a diverse panel of 17 sexually transmitted diseases (STDs)-associated pathogens including *Neisseria gonorrhoeae*, *Chlamydia trachomatis*, *Mycoplasma genitalium*, *Mycoplasma hominis*, *Ureaplasma*, Herpes simplex virus (HSV) types 1 and 2, and Human papillomavirus (HPV) types 6, 11, 16, 18, 31, 33, 35, 39, 54 and 58. The target genes are 16S rRNA gene for *N*. *gonorrhoeae*, *M*. *genitalium*, *M*. *hominism*, and *Ureaplasma*, the major outer membrane protein gene (*ompA*) for *C*. *trachomatis*, the glycoprotein B gene (*gB*) for HSV; and the *L1* gene for HPV. A total of 34 probes were selected for the microarray including 31 specific probes, one as positive control, one as negative control, and one as positional control probe for printing reference. The microarray is specific as the commensal and pathogenic microbes (and closely related organisms) in the genitourinary tract did not cross-react with the microarray probes. The microarray is 10 times more sensitive than that of the multiplex PCR. Among the 158 suspected HPV specimens examined, the microarray showed that 49 samples contained HPV, 21 samples contained *Ureaplasma*, 15 contained *M*. *hominis*, four contained *C*. *trachomatis*, and one contained *N*. *gonorrhoeae*. This work reports the development of the first high through-put detection system that identifies common pathogens associated with STDs from clinical samples, and paves the way for establishing a time-saving, accurate and high-throughput diagnostic tool for STDs.

## Introduction

The major causative agents of sexually transmitted diseases (STDs) are *Neisseria gonorrhoeae*, *Chlamydia trachomatis*, *Mycoplasma genitalium*, *Mycoplasma hominis*, *Ureaplasma*, Herpes simplex virus (HSV), Human papillomavirus (HPV), Human immunodeficiency virus (HIV), *Treponema pallidum* and *Trichomoniasis vaginalis* [1]. STDs are among the most common human infectious diseases and pose a major public health concern globally.

In 2008, there were an estimated 110 million prevalent STIs (sexually transmitted infection) among women and men in the United States. Of these, more than 20% of infections (22.1 million) were young people aged 15 to 24 years [2]. From 2004–2007 in China,the reported incidence of gonorrhea was declining, whereas that of syphilis,AIDS and the infection rates of HIV increased [3],During 2008–2012, the morbidity of STDs in Tianjin, P. R. China, showed an decreasing trend. The number of reported STD cases steadily decreases from 7,010 (incidence 62.87 per 10,000) in 2008 to 4,895 (incidence 36.14 per 10,000) in 2012 [4].

One of the most troublesome aspects of STD treatments is that STDs appear to be asymptomatic during the early infection stages, and the diseases resulting from different infectious agents often appear in similar symptom making clinical diagnoses difficult and unreliable in many cases [5]. Traditionally, microscopic examination and culturing of the pathogens are considered as the “gold standards” for the identification of the pathogens associated with STDs, and these procedures have been tailored to identify specific organisms, e.g., *N*. *gonorrhoeae* [6,7], *C*. *trachomatis* [7], *mycoplasmas*, *ureaplasmas* [8] and HSV [9]. However, the culture process takes three to seven days and sometimes even weeks to complete [10–12]. In addition, HPV and *T*. *pallidum* are difficult to be cultured [13,14].

Other strategies used in the detection of STD-associated pathogens include immunological assays and DNA amplification or hybridization [15,16]. Serological assays such as enzyme immunoassays (EIA) and/or direct immunofluorescence assays (DFA) provide rapid results and eliminate the need for cell culture-based assays, but these approaches sometimes do not rule out cross-reactive epitopes or distinguish between ongoing or past infections since antibody also responses to antigens that resulted from past infections [17]. Nucleic acid amplification is a very sensitive detection strategy, and can theoretically be used for the detection of as little as a few gene copies due to its high sensitivity. The commercial AMPLICOR CT/NG Amplification Kit licensed by Food and Drug Administration (FDA) of USA was used clinically to diagnose *C*. *trachomatis* and *N*. *gonorrhoeae* infections [18]. In China, PCR-fluorescence diagnostic kits have been used and certificated by the SFDA (State Food and Drug Administration, P. R. China, www.sfda.gov.cn) for STD diagnosis. However, each of these products is limited to the detection of only one or two pathogen(s) at one time. Although several systematic studies of microarray [19–21] have been conducted on the pathogens responsible for STDs, none of the studies is able to examine the most common clinical pathogens *N*. *gonorrhoeae*, *C*. *trachomatis*, *Ureaplasma*, *M*. *hominis*, *M*.*genitalium*, HSV and HPV simultaneously.

In this study, we introduce a multiplex, PCR-based oligonucleotide microarray to detect not only *N*. *gonorrhoeae*, *C*. *trachomatis*, *M*. *hominis*, *M*. *genitalium*, *Ureaplasma*, HSV, and HPV simultaneously, but also different serotypes of HSV types 1 and 2, and HPV types 6, 11, 16, 18, 31, 33, 35, 39, 54 and 58. The microarray can be used as a high throughput tool for screening sexually transmitted diseases.

## Materials and Methods

### Bacterial strains and clinical specimen collection

The 92 strains used for microarray specificity testing included 64 type strains and 28 clinical isolates (Table 1). Total five groups of 344 clinical specimens, including cervical and urethral swab specimens, genital warts, ulcers, and blister specimens, were obtained from three domestic hospitals of Tianjin, General Hospital of Tianjin Medical University, Tianjin Union Medical Center, and Changzheng Hospital in Tianjin, P. R. China. The clinical samples were kept in 500 μl of 150 mmol/L NaCl at -20°C. Out of the 344 clinical samples, the 158 suspected HPV specimens were used on the microarray.

**Table 1:** Strains used in this study.

Organisms	Number of strains and the source	Total number
Target strains used to test the specificity of the probes N = 24		
*Chlamydia trachomatis* types -A, B, E, F, G, H, J and L2	3^a^, 5^b^	8
*Human papillomavirus* types -6, 11, 16, 18, 31, 33, 35, 39, 54 and 58	10^b^ ^,^ ^c^	10
*Herpes simplex virus* types -1 and 2	2^b^ ^,^ ^c^	2
*Mycoplasma genitalium*	1^b^	1
*Mycoplasma hominis*	1^b^	1
*Neisseria gonorrhoeae*	1^d^	1
*Ureaplasma parvum* (serovars 3)	1^b^ ^,^ ^c^	1
*Ureaplasma urealyticum* (serovars 8)	1^b^ ^,^ ^c^	1
Other bacterial species used to test the specificity of the probes N = 68		
*Moraxella catarrhalis*	1^d^	1
*Candida albicans*	1^d^	1
*Citrobacter freundii*	1^b^	1
*Corynebacterium pseudodiphtheriticum*	1^b^	1
*Echerichia coli* O157, O149, O121 and O119	4^e^	4
*Enterococcus faecalis*	1^f^	1
*Enterococcus faecium*	1^a^, 2^g^	3
*Human papillomavirus* types -26, 34, 42, 43, 45, 51, 53, 55, 56, 59, 61, 62, 66, 67, 68, 74, 81, 82, 87 and 91	20^b^ ^,^ ^c^	20
*Klebsiella pneumoniae*	1^a^, 1^h^, 2^i^, 1^f^	5
*Lactobacillus acidophilus*	1^b^	1
*Mycoplasma hyorhinis*	1^j^	1
*Mycoplasma orale*	1^j^	1
*Mycoplasma pneumoniae*	1^b^	1
*Neisseria flavescen*	1^h^	1
*Neisseria lactamica*	1^h^	1
*Neisseria meningitidis*	1^d^	1
*Neisseria mucosa*	2^h^	2
*Neisseria sicca*	2^h^	2
*Neisseria subflava*	2^h^	2
*Proteus mirabilis*	2^h^	2
*Proteus vulgaris*	1^h^, 1^k^	2
*Pseudomonas aeroginosa*	3^f^, 2^i^	5
*Staphylococcus aureus*	3^i^, 1^f^	4
*Staphylococcus epidermidis*	1^a^	1
*Streptococcus agalactiae*	1^a^	1
*Streptococcus pyogenes*	3^i^	3

^a^, American Type Culture Collection (ATCC), USA.

^b^, Clinic isolate.

^c^, Plasmid or genome.

^d^, Microbe Test Center, Academy of Military Medical Sciences, China.

^e^, Institute of Medical and Veterinary Science (IMVS), Australia.

^f^, Institute of Microbiology, Chinese Academy of Sciences, China.

^g^, Institute of Food Fermentation industry, Ministry of Light Industry, China.

^h^, Czech Collection of Microorganisms (CCM), Czech Republic.

^i^, National Center for Medical Culture Collection (CMCC), China.

^j^, National Center for Veterinary Culture Collection (CVCC), China.

^k^, Culture Collection, University of Goteborg, Sweden.

### DNA extraction

Five hundred microlitres of each clinical sample were centrifuged at 15,000 *g* for 10 min, the pellets were resuspended in 100 μl of lysis buffer [50 mM NaOH, 0.5% NP-40, 0.5% Tween-20, 10 mM Tris-HCl (pH 8.0), 1 mM EDTA (pH 8.0), and 5% Chelex-100 (BioRad Corporation)], vortexed and boiled for 10 min. The lysates were centrifuged at 15,000 *g* for 5 min toremove the cell debris. A total of 3 μl of each supernatant containing extracted DNA were used as templates for PCR.

### Primer design

The 16S rRNA gene was used as the target gene for the detection of *N*. *gonorrhoeae*, *M*. *genitalium*, *M*. *hominism*, *Ureaplasma*; the major outer membrane protein gene (*ompA*) for the detection of *C*. *trachomatis*; the glycoprotein B gene (*gB*) for HSV; and the *L1* gene for HPV. The sequences of the target genes selected were obtained from GenBank. Related sequences were aligned using the CLUSTALX software. Based on the variable regions in the generated alignments, five compatible primer pairs for the multiplex PCR were designed using Primer Premier 5.0 software (Table 2). One primer pair for the amplification of the human β-globin gene [22] was selected as the positive control.

**Table 2:** Oligonucleotide primers used in the microarray assay.

Organism	Target gene	GenBank accession no.	Primer	Sequence (5’-3’)	Primer Conc (μM)	Amplicon size (bp)
*N*. *gonorrhoeae*	16S	EU233796	wl-5744	CGGAACGTACCGGGTAGC	0.2	648
			wl-5839	GCTACCCACGCTTTCGGA	0.2	
*M*. *hominis*, *M*. *genitalium*, *Ureaplasma*	16S	EU643797	wl-5967	GTAATACATAGGWYGCAAGCGTTATC	0.2	520
			wl-5968	CACCAYCTGTCAYWYBGWTAACCTC	0.2	
*C*. *trachomatis*	*ompA*	EU105066	wl-5963	ATCCTGCTGAACCAAGCCTTATGA	0.6	733
			wl-9516	CAYTCATGGTARTCAATAGAGGCA	0.6	
HSV	*gB*	M21629	wl-9940	AGCCCCTYCTCAGCAACACGC	0.3	407
			wl-10219	CGAGTTYTGSACGATCACGTTGTCC	0.3	
HPV	*L1*	X05015	wl-10247	AYHTGYAAATATCCWGATTA	0.9	260
			wl-10248	TGYARCCAATAWGGYTTATT	0.9	
Human	*β*-globin gene	AY260740	wl-9502	ACACAACTGTGTTCACTAGC	0.16	326
			wl-9503	CATCAGGAGTGGACAGATCC	0.16	

The degenerate base code is as follows: W = A or T, Y = C or T, R = A or G, S = C or G.

### Cloning

The target gene fragments of the selected organisms associated with respective STDs were amplified and cloned into the pGEM-T Easy vector (Promega, MA, USA), and transformed into competent *E*. *coli* DH5α cells. The clones were used to optimize the conditions for multiplex PCR to evaluate the sensitivity of the multiplex PCR and microarray assay and to screen the specificity of the probes.

### Target genes and optimization of multiplex PCR

Specific primer pairs for amplification of target genes were determined, including wl-5744 and wl-5839 for 16S rRNA sequence of *N*. *gonorrhoeae*, wl-5967 and wl-5968 for 16S rRNA sequence of *M*. *genitalium*, *M*. *hominis*, and *Ureaplasma*, wl-5963 and wl-9516 for *ompA* gene of *C*. *trachomati*, wl-9940 and wl-10219 for *gB* gene of HSV, wl-10247 and wl-10248 for *L1* gene of HPV, and wl-9502 and wl-9503 for the positive control β-globin gene, respectively.

Primer pair efficiency for six different groups of pathogens was determined based on the amplification of anticipated sizes by using different primer concentrations (0.10–0.90 μM). The primer concentration resulting in high-signal products was used subsequently, and they are 0.9 μM primers wl-10247 and wl-10248, 0.6 μM primers wl-5963 and wl-9516, 0.3 μM primers wl-9940 and wl-10219, 0.2 μM primers wl-5744, wl-5839, wl-5967 and wl-5968, and 0.16 μM primers wl-9502 and wl-9503 (Table 2).

### Two-step multiplex PCR and target gene labeling

The first PCR amplification step was carried out in a 25 μl reaction volume containing 1x PCR Buffer (70 mM KCl, 14 mM Tris-HCl, pH 8.3), 1.5 U Taq DNA polymerase (Sangon Corporation, Shanghai, China), 2.5 mM MgCl_2_, 400 μM each dNTPs, and primers at different concentrations (Table 2). The amplification was started at 80°C for 10 min then denatured at 95°C for 3 min followed by 35 cycles at 94°C for 45 s, 50°C for 2 min and 68°C for 1 min and a final step at 72°C for 10 min. An aliquot of 3 μl of PCR product was run on an agarose gel to confirm the size of the amplified DNA. The remaining reaction mixture was stored at -20°C.

For the second PCR step, the same PCR mixture was used except that 0.15 nM cyanine dye Cy3 (or Cy5)-dUTP (Amersham Biosciences UK Ltd., Little Chalfont, England) was included, the reverse primers were applied, and 3 μl of the PCR products from the first step were used as the template. The PCR conditions were the same as those described for the first PCR step. All labeled DNA samples were stored at -20°C in the dark.

### Oligonucleotide probe design

For each group or biotype of pathogens tested, 1–3 probes were designed using the OligoArray 2.0 based on GenBank sequences. One probe based on the human β-globin gene was designed as the positive control and one negative control probe and one positional reference and printing control probe were also used as described by Li et al., 2006 [23]. Each probe was 5’-amino-modified with a 16 poly(T)s tail followed by a stretch of specific sequence (synthesized by AuGCT Biotechnology Corporation, Beijing, China). All oligonucleotide probes are listed in Table 3.

**Table 3:** Oligonucleotide probes used in the microarray assay.

Organism	Target gene	GenBank accession no.	Probe	Sequence (5’-3’)
*N*. *gonorrhoeae*	16S	EU233796	OA-2331	CGTCATCGGCCGCCGATATTGGCAA
			OA-2332	TGCTTTCCCTCTCAAGACGTATGCGG
*C*. *trachomatis*	*ompA*	EU105066	OA-2102	CGAAAACAAAGTCWCCRTAGTAACC
			OA-2101	CATGCTGATAGCGTCAMMCCAAGTGG
HSV type 1	*gB*	M21629	OA-2334	GCGGCTCTGCTCTCGGAGGTGTTCC
			OA-2335	CGATGGCAACGCGGCCCAACATATC
HSV type 2	*gB*	EU018079	OA-2338	GCGCCCCAGCATGTCGTTCACGTGG
			OA-2339	CGACGGCGATGCGCCCCAGCATGTC
HPV type 6	*L1*	AF335604	OA-2312	CTACAGACGTKCGATTTCCACTACCC
			OA-2313	TTAACATATATACTACTCCCTACAG
HPV type 11	*L1*	M14119	OA-2314	TATTACCCCCTTTTACCAACAGGTC
			OA-2315	GTACATAAATACTACTAGCTACAGA
HPV type 16	*L1*	AF084952	OA-2316	TGTATAAATCGTCTGGTACATTTTC
			OA-2317	GGCTAAATTTGCAGTAGRCCCAGAG
HPV type 18	*L1*	X05015	OA-2318	ATAAGGATTGAGGCACAGTGTCACC
			OA-2319	GCTGCCAGGTGAAGCACGCATACCT
HPV type 31	*L1*	J04353	OA-2322	CAGTAGGGACCGATTCACCAACCGT
			OA-2323	AGTATGTACTGTTAGCTAAAGTAGC
HPV type 33	*L1*	M12732	OA-2324	ATCGGGAACAGCCTCTCCTAATGTA
			OA-2325	TGAATAGAGGCAGTAGTTCCTGAAC
HPV type 35	*L1*	M74117	OA-2327	AGTACTAGGCAATGTGCCAGTGGTA
HPV type 39	*L1*	M62849	OA-2655	CAGAACTACCGGTGTTTGCACGT
			OA-2659	TATACAATTGGGCAGGAATGGCGTC
HPV type 54	*L1*	U37488	OA-2330	ACTGTCARGGTTACCTGAGGATTTC
HPV type 58	*L1*	D90400	OA-2321	AAATGCACTACTTTGGATAACTGCA
*M*. *hominis*	16S	AF275263	OA-2055	TTGGATACTAGCAAACTAGAGTTAGA
			OA-2056	AGTCTGGAGTTAAATCCCGGGGCTCAAC
*Ureaplasma*	16S	AF073455	OA-2062	GCTCGAACGAGTCGGTGTTGTAGCT
*M*. *genitalium*	16S	AY466443	OA-2051	TGTCGGAGCGATCCCTTCGGTAGTGAA
			OA-2052	TACTAGCTGTCGGAGCGATCCCTTC
			OA-2053	GCATTGGAAACTATCAGTCTAGAGTGT
Positive control	*β*-globin gene	AY260740	OA-2340	CTCTTGGGTTTCTGATAGGCACTGA
Negative control			Neg	TTTTTTTTTTTTTTTTTTTTTTTTTTTTTTTTTTTTTTT
Positional control			Cy3	TTTTTTTTTTTTTTTTTTTTTTTTTTTTTTTTTTTTTTTT-Cy-3’

The degenerate base code is as follows: W = A or T, Y = C or T, R = A or G, S = C or G, K = G or T, M = A or C.

### DNA array preparation

**Fig 1:**
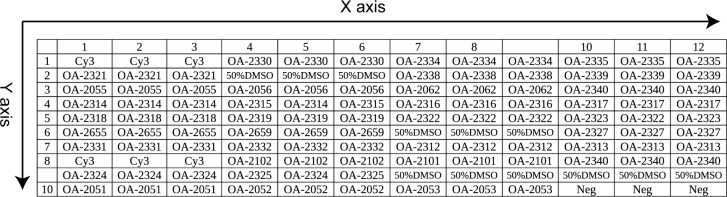
Probe positions on the microarray. Cy3 is the positional reference and printing control probe; the OA-2340 is the positive control probe; Neg is the negative control probe; and the others are the specific probes for the target pathogens.

The probes were dissolved in 50% dimethyl sulfoxide (DMSO) to a final concentration of 1 μg/μl and printed onto aldehyde group-modified glass slides (CEL Corporation, USA) using a SpotArray72 (Perkin-Elmer Corporation, CA, USA). Each probe was spotted in triplicate and each slide consisted of six microarrays framed with a 12 μl Geneframe (Beijing Capital Biochip Corporation, Beijing, China) which constituted individual reaction chambers. A schematic diagram of the probe positions of the single microarray is shown in Fig 1.

### Hybridization process

The hybridization was performed as follows: a volume of 8 μl of labeled target single-stranded PCR products were mixed with 8 μl of hybridization buffer {25% formamide, 0.1% sodium dodecyl sulfate, 6x SSPE (1x SSPE contains 0.18 M NaCl, 10 mM NaH_2_PO_4_, and 1 mM EDTA, pH 7.7)}, and the mixture was applied to a hybridization chamber and incubated at 50°C for 1.5 h in a water bath. After incubation, the slide was washed sequentially in solution A (1x SSC, 0.2% SDS) for 3 min, solution B (0.2x SSC) for 3 min and solution C (95% alcohol) for 1.5 min. The slide was dried under a gentle air stream before it was scanned.

### Data acquisition and analysis

Slides were scanned with laser beam at 532 nm for Cy3 (or 635 nm for Cy5) nm using the 4100A biochip scanner (Axon Corporation, CA, USA). A positive detection result was reported when all the probes of the given target gene generated hybridization signals above the signal-to-noise-ratio threshold (3.0).

### Nucleotide sequence and microarray accession numbers

The microarray dataset was deposited into the Gene Expression Omnibus database under the accession number GSE69508.

## Results

### Amplicons of target genes

The size of the amplicons, ranged from 260–733 base pairs (bp) in length, of the pathogens are as follows: *C*. *trachomati*, 733 bp; *N*. *gonorrhoeae*, 648 bp; *M*. *genitalium*, *M*. *hominis*, and *Ureaplasma*, 520 bp; HSV, 407 bp; HPV, 260 bp; and β-globin, 326 bp. The results indicated that the primers were specific for the respective target genes and compatible in the multiplex PCR reaction.

### Probe screening and specificity

**Fig 2:**
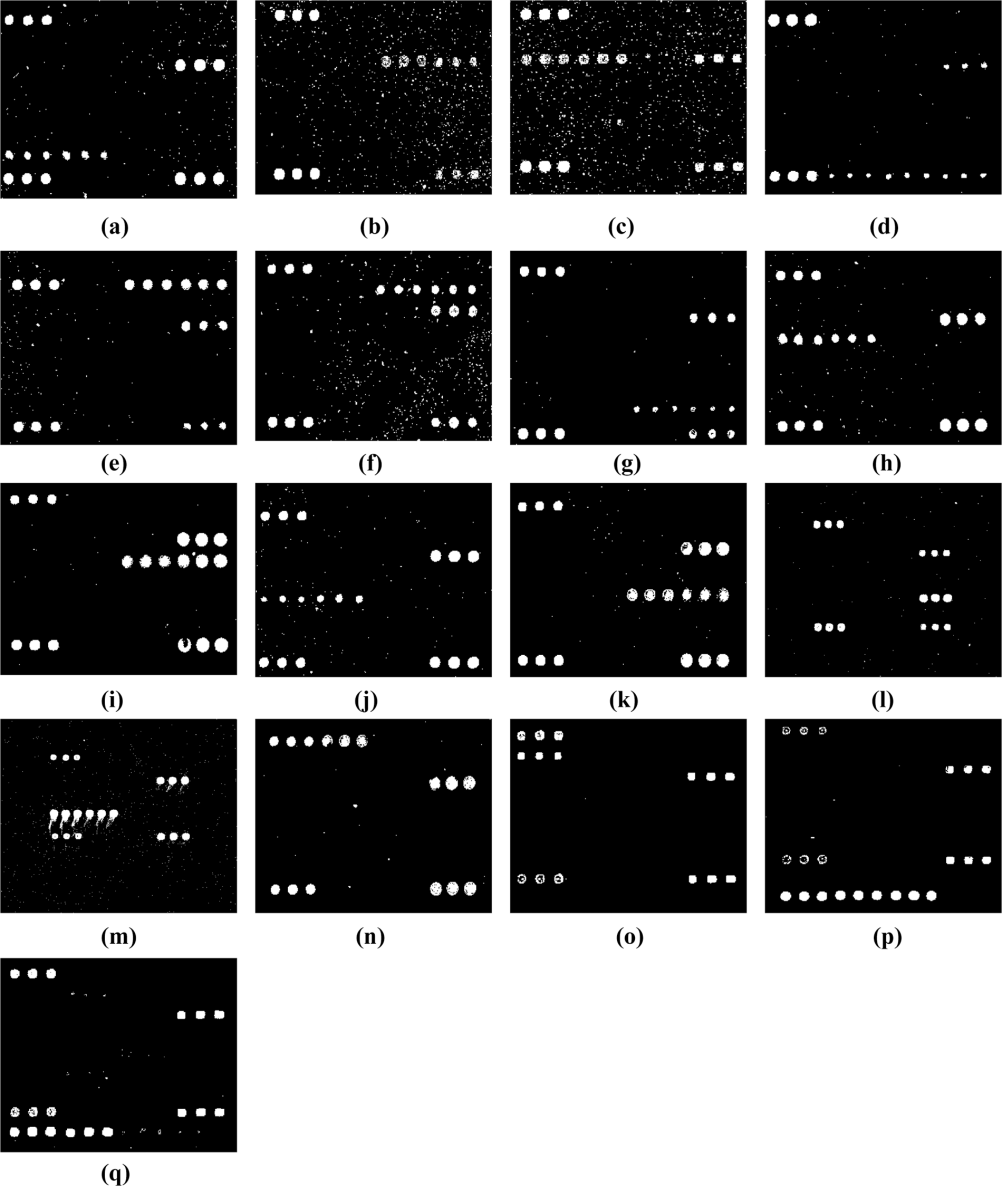
Hybridization patterns of different pathogens. (a) *N*. *gonorrhoeae*, (b) *Ureaplasma*, (c) *M*. *hominis*, (d) *C*. *trachomatis*, (e) HSV type 1, (f) HSV type 2, (g) HPV type 6, (h) HPV type 11, (i) HPV type 16, (j) HPV type 18, (k) HPV type 31, (l) HPV type 35, (m) HPV type 39, (n) HPV type 54, (o) HPV type 58, (p) *M*. *genitalium*, and (q) HPV type 33.

For each sample, at least three hybridization reactions were replicated to demonstrate the reproducibility of the microarray method. The DNA microarray was tested using 24 strains of the target species and 68 strains of commensal microbes that are either common to the genitourinary tract or genetically close related to the target species (Table 1). From 85 oligonucleotide probes initially tried in the microarray, 34 probes were selected as suitable for the microarray including 31 probes for specific genes, one as positive control probe, one as negative control probe, and one as positional control probe for printing reference (Table 3). The hybridization results for different pathogens are shown in Fig 2.

The microarray specifically identified the 17 target strains. *N*. *gonorrhoeae* produced positive signals with its specific probes of OA-2331 and OA-2332, as well as the positive control probe OA-2340 and the positional and printing control probe Cy3 but not with the other probes (Fig 2a). Similarly, *Ureaplasma* produced positive signals with its specific probes of OA-2062 (Fig 2b); *M*. *hominis*, with OA-2055 and OA-2056 (Fig 2c); *C*. *trachomatis*, with OA-2102 and OA-2101 (Fig 2d); HSV type 1, with OA-2334 and OA-2335 (Fig 2e); HSV type 2, with OA-2338 and OA-2339 (Fig 2f); HPV type 6, with OA-2312 and OA-2313 (Fig 2g); HPV type 11, with OA-2314 and OA-2315 (Fig 2h); HPV type 16, with OA-2316 and OA-2317 (Fig 2i); HPV type 18, with OA-2318 and OA-2319 (Fig 2j); HPV type 31; with OA-2322 and OA-2323 (Fig 2k); HPV type 35, with OA-2327 (Fig 2l); HPV type 39, with OA-2655 and OA-2659 (Fig 2m); HPV type 54, with OA2330 (Fig 2n); HPV type 58, with OA-2321 (Fig 2o); *M*. *genitalium*, with OA-2051, OA-2052, and OA-2053 (Fig 2p); and HPV type 33, with OA-2324 and OA-2325 (Fig 2q).

Multiplex PCR assays carried out with DNA from unrelated organisms (Table 1) did not amplify products except when *Escherichia coli*, *Enterococcus faecalis*, *Enterococcus faecium*, *Klebsiella pneumoniae*, *Citrobacter freundii*, and *Mycoplasama pneumoniae* were used as templates, however, none of these products hybridized to the microarray probes. None of the strains closely related to the target species, except *M*. *pneumoniae* which shares 98% homology of the 16S rRNA sequence with *M*. *genitalium*, hybridized to the specific probes on the microarray. It was reported that *M*. *pneumoniae* are rarely associated with genitourinary infections [8].

### Detection sensitivity

**Fig 3:**
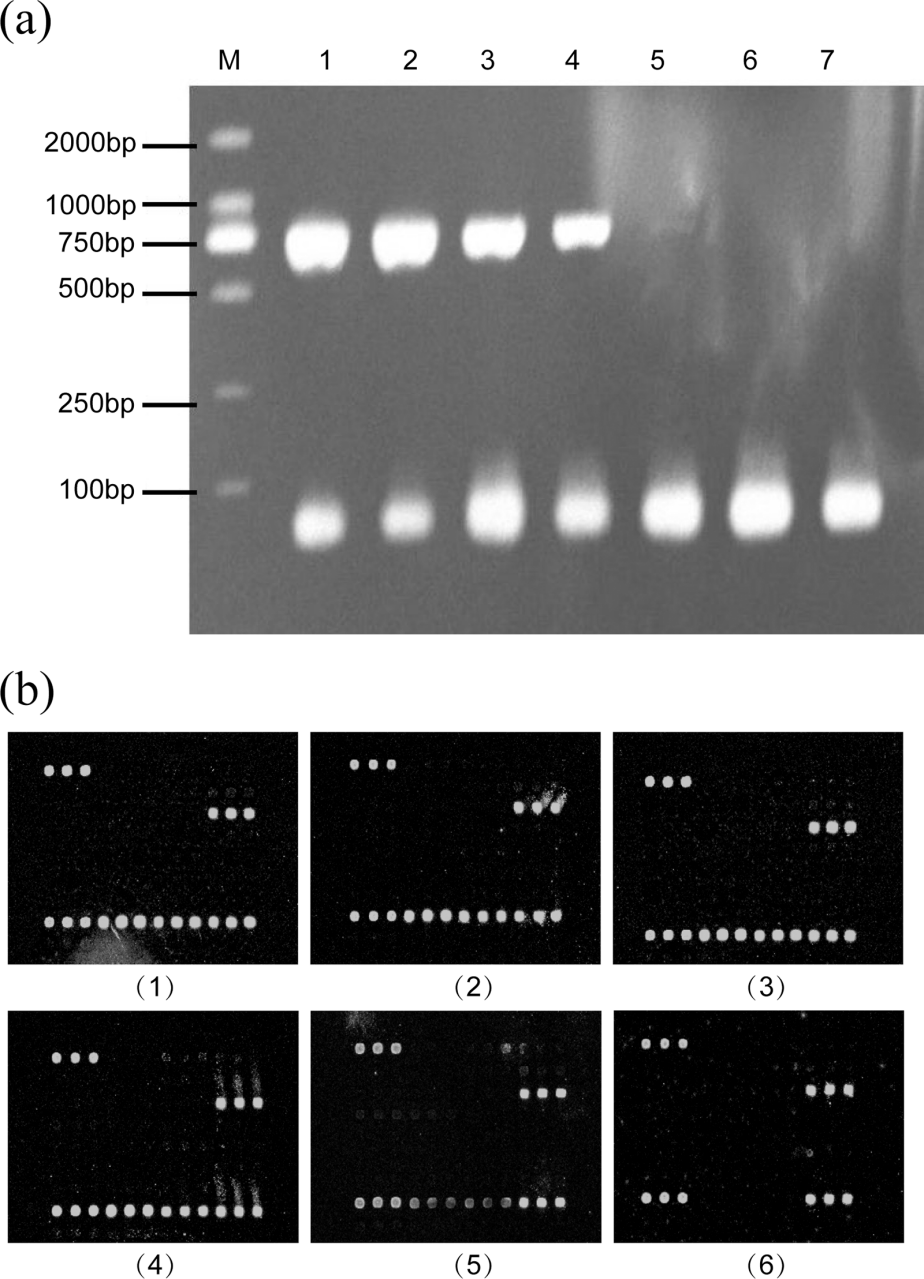
Detection sensitivity of *C*. *trachomatis*.(a)Detection sensitivity of *C*. *trachomatis* with the multiplex PCR. (1) 10^7^ copies, (2) 10^6^ copies, (3) 10^5^ copies, (4) 10^4^ copies (5) 10^3^ copies, (6) 10^2^ copies, and (7) 10^1^ copies (b)Detection sensitivity of C. trachomatis with the microarray. (1) 10^6^ copies, (2) 10^5^ copies, (3) 10^4^ copies (4) 10^3^ copies, (5) 10^2^ copies, and (6) 10^1^ copies.

Ten-fold serial dilutions, ranging from 10^0^−10^7^ copies of target DNA obtained from *N*. *gonorrhoeae*, *C*. *trachomatis*, *Ureaplasma*, HSV type 2 and HPV type 11 in addition to the human β-globin gene, were used to test the sensitivity of the multiplex PCR and the microarray. The sensitivity of the multiplex PCR was at 10^3^–10^4^ copies and that of the microarray at 10^2^–10^3^ copies for *C*. *trachomatis*. The microarray is 10 times more sensitive than that of the multiplex PCR (Fig 3a and 3b).

### Analysis of clinical samples using the microarray

Among the 158 suspected HPV specimens, the positive signals generated by the microarray showed that 49 samples contained HPV, 21 samples contained *Ureaplasma*, 15 contained *M*. *hominis*, four contained *C*. *trachomatis*, and one contained *N*. *gonorrhoeae*. Out of the 49 HPV positive samples, 24 are positive for one HPV type, and the other 25 are positive for more than two HPV types. The frequency of HPV types 6、11、16、18、31、33、35、39、54 and 58 detected are 13、7、15、4、4、6、4、4、3 and 5, respectively.

## Discussion

Primers and probes specific to STD pathogens were designed for the microarray. The *N*. *gonorrhoeae*-specific primers were based on the 16S rRNA gene sequence, which is a more specific than the one described by Farrell [24], since the primers in this study did not generate any amplicons from closely related *N*. *gonorrhoeae* strains. The primers used for the detection of HPV, *Mycoplasma* and *Ureaplasma* were also improved for specificity from those described by Jenkins and Yoshida [8,25]. The primers designed for the detection of *C*. *trachomatis* and HSV were designed from specific sequences encoding the major outer membrane protein and glycoprotein B, respectively [6,26,27]. The specificity of this microarray was further enhanced by the pathogen-specific probes included in the chip.

The most difficult problem associated with multiplex PCR was the uneven amplification of targeting products, leading to poor sensitivity. In order to overcome the problem, the multiplex PCR parameters, including the primer concentrations, the PCR buffer, and the annealing time and temperatures, were optimized to ensure the sensitivity of optimized multiplex PCR equals to that of conventional PCR. Following the optimized multiplex PCR analysis, single-strand primer extension was employed to fluorescently label respective products. This two-step method improved the hybridization efficiency and reliability.

It is important to employ an internal control to verify DNA extraction efficiency from the samples and performance of all of the components in the reaction [28]. In our system, amplification of the human β-globin gene verified the efficiency of the DNA extraction and hybridization. Inability to amplify the human β-globin gene product or to detect the corresponding fluorescence signal indicates that the DNA concentration in the sample was too low or that the inhibitors were present in the reactions.

The microarray is able to detect the presence of STDs related 17 pathogens, *N*. *gonorrhoeae*, *C*. *trachomatis*, *M*. *hominis*, *M*. *genitalium*, *Ureaplasma*, HSV types 1 and 2, and HPV types 6, 11, 16, 18, 31, 33, 35, 39, 54 and 58 simultaneously within 7.5 hours from DNA preparation. As the data showed in Fig 2, the assay has differentiated successfully 17 pathogens based on the specificity of the probes applied with 10 times better resolution than that of the multiplex PCR. Compared to the traditional methods, the high throughput microarray assay has a number of advantages, First, it is particularly useful for the diagnosis of multiple infections simultaneously; second, it could be used to detect not only bacterial pathogens, but *Chlamydia*, *Mycoplasma*, *Ureaplasma*, and viruses (HSV and HPV) as well; trd, it is able to inspect the most prevalence of serogroups of HPV in the samples; and fourth,it is very unlikely to get false positive results, as this technology requires two specific steps, first, PCR reaction using specific primers, and second, hybridization applying specific probes. The methodology underlying in this microarray provides a general mean to detect STD pathogens and could be applied in other diagnosis.

This study offers a high throughput, low cost diagnosis tool, alternative to conventional, time-consuming, labor-intensive methods, to detect STDs related pathogens quickly and reliably. The major limitation of the study is that we need to expand the probes to include all the STDs pathogens, adding HIV and *Treponema pallidum*. In conclusion, the development of a new multiplex PCR-based microarray assay for a high-throughput platform for the detection of multiple infectious agents from multiple samples simultaneously is presented. These data suggested that the microarray analysis developed is a reliable, sensitive, and specific approach for the diagnosis of STD-associated pathogens.
